# A *Legionella pneumophila* amylase is essential for intracellular replication in human macrophages and amoebae

**DOI:** 10.1038/s41598-018-24724-1

**Published:** 2018-04-20

**Authors:** Ashley Best, Christopher Price, Mateja Ozanic, Marina Santic, Snake Jones, Yousef Abu Kwaik

**Affiliations:** 1 Department of Microbiology and Immunology, College of Medicine, University of Louisville, Louisville, KY USA; 2 Department of Microbiology and Parasitology, Faculty of Medicine, University of Rijeka, Rijeka, Croatia; 3 Center for Predictive Medicine, University of Louisville, Louisville, KY USA

## Abstract

*Legionella pneumophila* invades protozoa with an “accidental” ability to cause pneumonia upon transmission to humans. To support its nutrition during intracellular residence, *L*. *pneumophila* relies on host amino acids as the main source of carbon and energy to feed the TCA cycle. Despite the apparent lack of a requirement for glucose for *L*. *pneumophila* growth *in vitro* and intracellularly, the organism contains multiple amylases, which hydrolyze polysaccharides into glucose monomers. Here we describe one predicted putative amylase, LamB, which is uniquely present only in *L*. *pneumophila* and *L*. *steigerwaltii* among the ~60 species of *Legionella*. Our data show that LamB has a strong amylase activity, which is abolished upon substitutions of amino acids that are conserved in the catalytic pocket of amylases. Loss of LamB or expression of catalytically-inactive variants of LamB results in a severe growth defect of *L*. *pneumophila* in *Acanthamoeba polyphaga* and human monocytes-derived macrophages. Importantly, the *lamB* null mutant is severely attenuated in intra-pulmonary proliferation in the mouse model and is defective in dissemination to the liver and spleen. Our data show an essential role for LamB in intracellular replication of *L*. *pneumophila* in amoeba and human macrophages and in virulence *in vivo*.

## Introduction

The accidental human pathogen, *Legionella pneumophila*, causes an atypical pneumonia when water droplets, stemming from a contaminated water source such a cooling tower or humidifier, are inhaled by humans, which are considered as accidental host^1–3^. Over 20 protozoa species are known to harbor *Legionella* species, likely with more yet to be identified^4^. Growth within the natural protozoan host serves as a “training grounds”, priming for infection of human alveolar macrophages, as these bacteria are more infectious than their free-living counterparts^5–7^. Success in replicating in macrophages may have been facilitated by the exploitation of evolutionarily conserved host processes, which allow *L*. *pneumophila* to modulate conserved pathways in both macrophages and protozoa^4,8–10^. Inhaled bacteria enter into the lungs where they primarily reside and proliferate within alveolar macrophages^11–13^. The intracellular lifecycle in the evolutionarily distant host cells is nearly identical^4^. Once the bacterium enters the host cell, it actively evades lysosomal fusion and intercepts ER-derived secretory vesicles to generate and ER-derived vacuole, known as the *Legionella*-containing vacuole (LCV)^14–17^, and modulate a plethora of cellular and innate immune processes^18–21^.

Essential to intracellular replication is the Dot/Icm Type 4b secretion system (T4SS), which inject proteins, known as “effectors”, from the bacterium to the host cytoplasm to modulate host processes^22–24^. Because of the broad host range, *L*. *pneumophila* has evolved over 320 effectors that are translocated by the Dot/Icm system and utilized as a “toolbox” to modulate cellular processes of various environmental hosts^25–29^. Many unique mechanisms of interfering with host processes, such as lysosomal-evasion and trafficking, have been identified and attributed to specific Dot/Icm effectors^23,25,30–33^. The effector’s ability to interfere with the function of eukaryotic host target proteins, comes from their evolutionary history; many effectors are derived from eukaryotic proteins acquired by inter-kingdom horizontal gene transfer (HGT)^26,34–36^.

The primary food source for *L*. *pneumophila* is amino acids which are used for carbon and energy through feeding the TCA cycle^37–40^.The generation of host amino acids by *L*. *pneumophila* is an effector-driven process^41^. Substantial generation of host amino acids is required in human macrophages and amoebae where the effector AnkB hijacks the host ubiquitin-proteasome protein degradation machinery, which is required for successful pathogen replication in the host^41–44^. In contrast to human macrophages and amoebae, during infection of mouse macrophages with the *L*. *pneumophila* LP02 strain, the mammalian target of rapamycin complex 1 (mTORC1), a nutrient/energy sensor, is inhibited by multiple effectors to prevent protein synthesis, thus liberating amino acids for bacterial consumption^45^. Distinct pathogen mechanisms of generating host cell amino acids may be employed within diverse host cells and the pathogen mechanism may differ by various strains of *L*. *pneumophila* to acquire the high levels of host amino acids needed for replication.

Glucose is minimally metabolized by *L*. *pneumophila* through the Entner-Doudoroff (ED) pathway^39,46–48^. Traditional glycolysis through the Embden-Meyeroff-Parnas (EMP) pathway is also minimal, despite all the necessary genes being present in the *L*. *pneumophila* genome^39,46^. Glucose does not support growth of *L*. *pneumophila*, but it is predominantly imported by *L*. *pneumophila* upon termination of growth^39,46^, as the bacterium is preparing for cellular egress, and used mainly for the generation of the storage molecule, poly-3-hydroxybutyrate (PHB) through the ED pathway^46,49^. During nutrition deprivation, PHB is converted to acetyl-CoA that feeds the TCA cycle^50–52^. Genes involved in glucose metabolism and glucose uptake are up-regulated during growth in amoebae and may play a role in infection^52,53^.

Amylases are a conserved group of enzymes that catalyze hydrolysis of starch and glycogen into glucose^54^. They are members of a larger family, called glucosidases, and include other enzymes such as cellulase and lactase^55^. Interestingly, despite the minimal need of glucose by *L*. *pneumophila*, four putative amylases have been identified in the *L*. *pneumophila* genome, Lpg0422, Lpg1669, Lpg1671, and Lpg2528. The Lpg0422 (GamA) enzyme is the only characterized amylase^56^. It is secreted by the Type II secretion system (T2SS) and expressed during exponential growth but not required for intracellular growth^53,56^. Lpg1669 is a putative amylase that lacks putative secretion signals for the T4SS or T2SS, based on bioinformatical analysis. Lpg1671 is predicted to be a T4SS substrate but its role in intracellular infection is not known^57,58^. The gene for *gamA* is found in most *Legionella* species and *lpg1669* and *lpg1671* are found in three species of *Legionella*.

The predicted putative amylase, Lpg2528, has been designated as LamB. Among the 60 *Legionella* species, *L*. *pneumophila* and *L*. *steigerwaltii* are the only two *Legionella* species to harbor *lamB*. Since *L*. *pneumophila* is responsible for 85% of Legionnaire’s disease cases, we decided to determine the role of LamB in the intracellular infection of amoebae and human macrophages^2,59^. Here we show that despite the minimal role of glucose in *L*. *pneumophila* metabolism, the LamB amylase is surprisingly necessary for intracellular replication in amoebae and human macrophages, and is required for virulence *in vivo*, in the A/J mouse model.

## Results

### Identification of amylases in *L*. *pneumophila*

**Figure 1:**
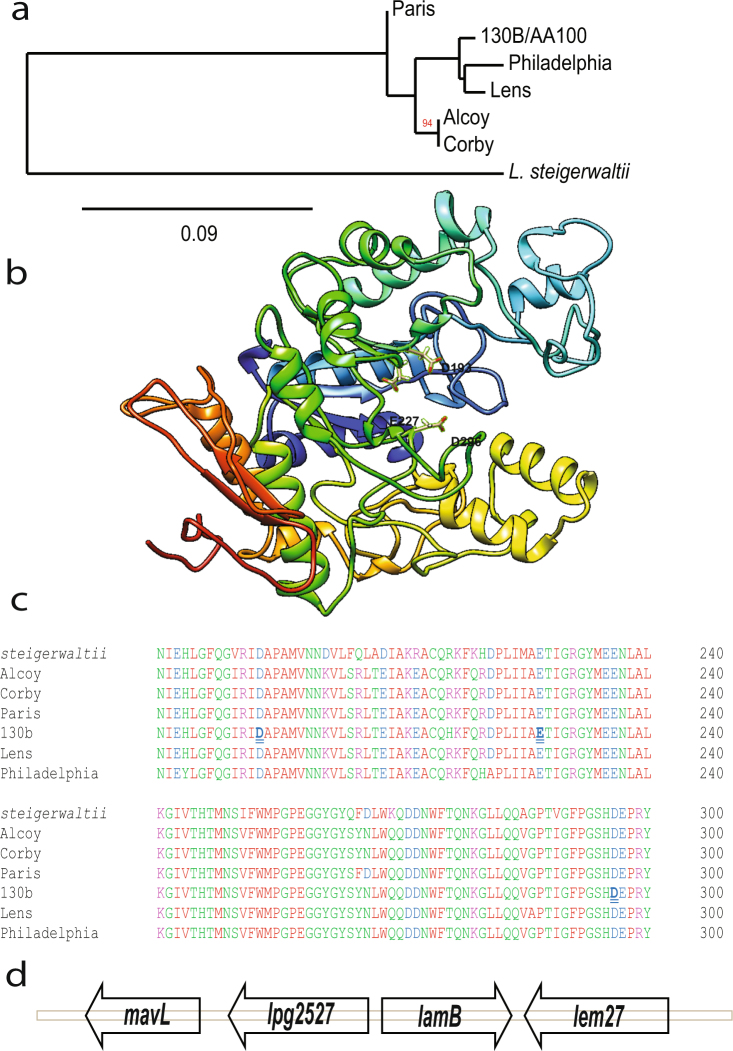
LamB is a putative amylase unique to *L*. *pneumophila*. (**a**) Phylogram representation of LamB divergence in *L*. *pneumophila* strains and *L*. *steigerwaltii*. Measure of node support was determined by aLRT using Phylogeny.fr (**b**) LamB is conserved among *L*. *pneumophila* strains and is only found in one other *Legionella* species, *L*. *steigerwaltii*. (**c**) The structure of LamB, generated from I-TASSER server, which suggests it is an amylase^96^. Highlighted within the catalytic binding pocket of amylases are resides critical for catalytic activity, D193, E227, and D296. The *lamB* gene is found on a monocistronic operon within the *L*. *pneumophila* genome, this area of the chromosome is shown in (**d**).

Based on domain sequence homology, three new putative amylases were identified in the *L*. *pneumophila* genome, in addition to the one described (GamA) amylase (Fig. S1a–c)^56,60^. The Lpg2528 putative amylase is designated as LamB, which is encoded by a monocistronic gene (Fig. 1d). Considering LamB is only present in *L*. *pneumophila* and *L*. *steigerwaltii*, of the 60 *Legionella* species, it is more likely that LamB has been acquired after the speciation event of *L*. *pneumophila* and suggests that *L*. *steigerwaltii* may have arisen recently from *L*. *pneumophila* (Fig. 1a). The evolution of this gene in *L*. *pneumophila* mirrors that of the strain evolution (Fig. 1a). *L*. *pneumophila* strain Lens is most related in genome sequence homology to strain 130b/AA100, which is seen with *lamB* (Branch length, 99)^61^. Similarly, *L*. *pneumophila* strain Alcoy is most homologous to strain Corby, as also seen with *lamB* (Branch length, 94)^61^. LamB shares amino acids sequence homology only with other soil and freshwater organisms such as, *Methylobacterium* and *Insolitispirillum* (see Supplementary Fig. S2). Thus, it is likely that *lamB* may have been acquired by HGT from other intra-amoebal or planktonic, environmental organisms. Because *L*. *pneumophila* is responsible for 85% of Legionnaire’s disease cases, we characterized the role of this enzyme in the intracellular infections of human monocyte-derived macrophages (hMDMs) and *A*. *polyphaga*^2,59^.

### Structure of LamB and its potential secretion

LamB has an α-amylase domain (residues 18–376) that is structurally similar to the crystalized glucosidase of *Streptococcus mutants*, SmDG (Fig. S1d)^62,63^. The putative catalytic site, which is located within the predicted catalytic pocket of the enzyme, is conversed amongst amylases and in LamB of *L*. *pneumophila* and *L*. *steigerwaltii* (Figs 1b,c and S1d). Iterative Threading Assembly Refinement (I-TASSER) is a bioinformatics method of predicting the three-dimensional structure of proteins based on fold recognition^64,65^. The server also predicts ligand binding sites, and gene ontology. Structural modeling of LamB using the I-TASSER database shows structural similarity with other crystalized glucosidases (Fig. 1b).

*L*. *pneumophila* proteins can access the host cytosol by two major routes, translocation via the T4SS or through secretion into the LCV lumen by the type II secretion system (T2SS) and into the cytosol through the semipermeable LCV membrane^22,66^. *L*. *pneumophila* secretes many proteins via the T2SS, which is critical for intracellular growth and pulmonary disease^67–69^. Twenty proteins were identified to be secreted by the T2SS of *L*. *pneumophila* while an additional 250+ proteins have been suggested to contain a putative T2SS signal^70^. LamB was not identified by any of these methods as potential type-II substrate^70^. In addition, LamB lacks the N-terminal secretion signal characteristic of T2SS substrates^69^.

**Figure 2:**
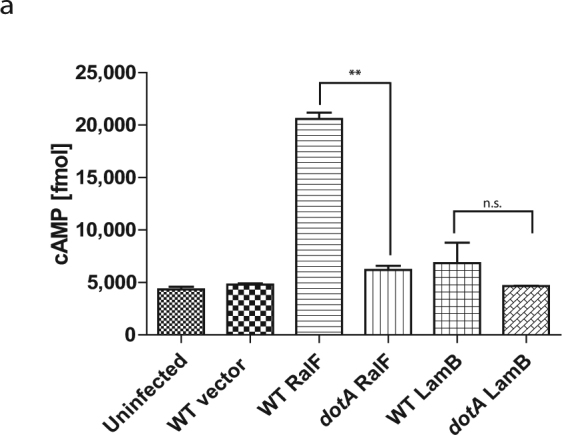
CyaA-LamB reporter is not translocated by the Dot/Icm T4SS. (**a**) Adenylate cyclase fusions of LamB expressed in *L*. *pneumophila* and infected into hMDMs for 1 hr, in triplicate, using known T4SS effector, RalF, as a positive control. Production of cAMP was assessed by ELISA. Data is shown as mean cAMP concentration ± SD, n = 3 independent infections.

In order to be translocated by the Dot/Icm translocation system, effectors are recognized by a translocation signal on the C-terminus; alterations at the C-terminus of effectors causes a failure in translocation^57,71,72^. However, no translocation consensus sequence exists for all effectors of the Dot/Icm translocation system. Machine learning techniques have identified conserved bi- and tri-residues at the C-terminal end of *L*. *pneumophila* effector proteins, 10% of known effectors do not contain at any of these motifs and some proteins harboring these motifs are not translocated effectors^57,73^. LamB contains seventeen bi-residues identified to be heavily enriched in the last 100 amino acids of the C-terminus of T4SS effector proteins (see Supplementary Fig. S3)^73^. To determine whether LamB was Dot/Icm-translocated, the adenylate cyclase (CyaA) reporter function was used^74,75^. Transformation of plasmids expressing reporters CyaA-LamB or the positive control, CyaA-RalF, as fusion proteins was performed in WT *L*. *pneumophila* and the translocation-deficient mutant, *dotA*. The data showed that the CyaA-LamB reporter was not translocated by the Dot/Icm translocation system, as there was no significant difference between the secretion of CyaA-LamB by WT *L*. *pneumophila* or the *dotA* mutant (Student *t*-test, *p* > 0.2); whereas, the control, CyaA-RalF, was readily translocated into the host cells during infection with WT *L*. *pneumophila* but not the *dotA* mutant (Student *t*-test, *p* < 0.01) (Fig. 2).

### The amylase activity of LamB

**Figure 3:**
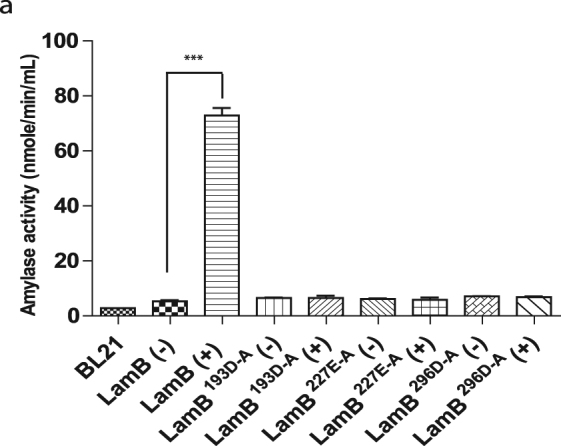
LamB is a functional amylase. (**a**) Amylase activity of ITPG-inducible (+), GST-LamB fusions and catalytic site mutants, expressed in *E*. *coli*, was assessed by colormetric assay of cleavage of an artificial compound. Data are representative of three independent experiments represented by mean amylase activity, of triplicate sample, as measured by cleavage of ethylidene-pNP-G7 into *p-*nitrophenol, shown as mean amylase activity ± SD, n = 3 independent cultures.

To confirm the putative enzymatic activity of LamB as an amylase, *in vitro* biochemical activity was determined by using the standard amylase activity colorimetric assay, quantifying the cleavage of ethylidene-pNP-G7 to *p-*nitrophenol, which can be measured at 405 nm^76,77^. Three highly conserved residues, D193, E227, and D296 were identified in the catalytic pocket of LamB and confirmed with domain alignment to other amylases (Fig. 1b,c). Constructs harboring native LamB or three LamB variants of single amino acid substitutions in the catalytic pocket were expressed in *E*. *coli* as GST fusion proteins, controlled by an IPTG inducible promotor. Expression of these proteins was confirmed by western blot (Fig. S4). With IPTG induction, amylase activity was highest for the wild type protein compared to uninduced (Student *t*-test, *p* < 0.001) (Fig. 3). The three catalytic mutants showed no amylase activity after inducing with IPTG. These data confirmed that LamB is indeed an amylase and that the identified catalytic pocket is essential for enzymatic activity.

### Requirement of LamB for growth in amoebae and hMDMs

In order to test the role of LamB in intracellular growth, a *lamB* null mutant was generated. Complementation was achieved by expression of *lamB* on a plasmid, *lamB*/C. The LamB variants with amino acid substitutions in the catalytic domain of LamB, D193A, E227A, and D296A were also introduced into the *lamB* null mutant (Fig. 1c). Infections of *Acanthamoeba polyphaga* or hMDMs were performed, as we previously described^78^.

**Figure 4:**
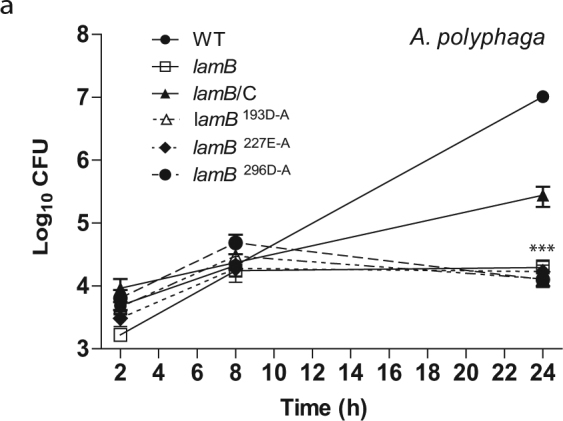
LamB is required for growth in amoebae. To determine intra-vacuolar replication of the WT strain, the *dotA* mutant, the *lamB* mutant, catalytic mutants (D193A, E227A, and D296A), and complemented *lamB* mutant (*lamB/*C), *A*. *polyphaga* were infected and number of CFUs were determined at 2, 8, and 24 h post-infection. Data points represent (mean CFUs ± SD, n = 3) and are representative of three independent experiments.

**Figure 5:**
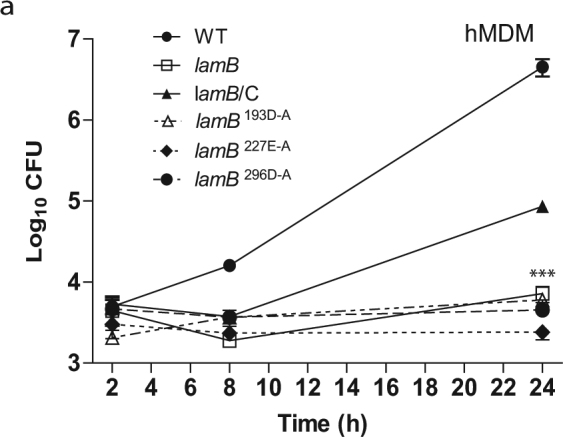
LamB is required for growth in hMDMs. To determine intra-vacuolar replication of the WT strain, the *dotA* mutant, the *lamB* mutant, catalytic mutants (D193A, E227A, and D296A), and complemented *lamB* mutant (*lamB/*C), hMDMs were infected and number of CFUs were determined at 2, 8, and 24 h post-infection. Data points represent (mean CFUs ± SD, n = 3) and are representative of three independent experiments.

The *lamB* mutant was severely defective for intracellular growth in hMDMs and *A*. *polyphaga* (Figs 4 and 5). At 24 h post-infection, there was a significant difference in the replication of the null mutant and the three catalytically inactive mutants compared to WT *L*. *pneumophila* (Two-way ANOVA, *p* < 0.001). The growth defect was partially restored to the mutant by in trans-complementation of the gene, which is likely due to loss of the plasmid. However, complementation of the null mutant with any of the three catalytic variants did not restore any growth to the *lamB* mutant in *A*. *polyphaga* or hMDMs (Figs 4 and 5). This defect is not attributed to a growth defect *in vitro*, as the *lamB* mutant grows just as well as the WT strain in broth (see Supplementary Fig. S5). These data show that LamB is necessary for intracellular replication of *L*. *pneumophila* in both hMDMs and *A*. *polyphaga*. Indeed, it is the amylase activity of LamB that contributes to its essential role in intracellular growth, indicating the requirement for degradation of polysaccharides by *L*. *pneumophila*. This is surprising, considering the minimal role of glucose in metabolism of *L*. *pneumophila*, and that it is mainly utilized during late stages of growth to synthesize the PHB storage compound.

To determine if generation of host glucose by LamB was necessary for intracellular replication, *A*. *polyphaga* was supplemented with exogenous glucose during infection (Fig. S6). Glucose supplementation did not rescue the *lamB* mutant for its defect in intracellularly replication nor did it alter the growth of the WT strain or the complemented mutant (Fig. S6).

### Role of LamB *in vivo*

**Figure 6:**
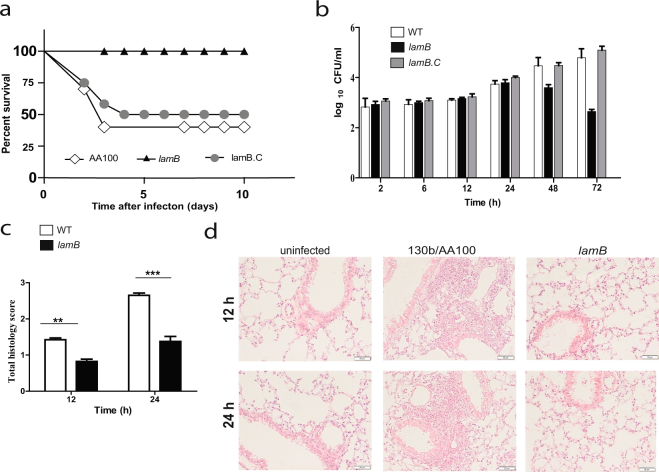
Role of LamB in virulence in A/J mice. Mice were infected intra-trachaelly with 10^6^ CFUs of WT (white), the *lamB* mutant (black), or the completed, *lamB/*C (grey). (**a**) Survival of the mice over days and (**b**) CFU organ burden in the lungs was assessed at the various time points. Pulmonary histopathology scores at 12 h (Student *t*-test, *p* < 0.01) and 24 h (Student *t*-test, *p* < 0.005) are shown in (**c**) and representative images of uninfected, WT, and *lamB* are shown in (**d**).

Given that *A*. *polyphaga* and hMDMs restrict the *lamB* mutant, we sought to determine the role of LamB in intrapulmonary growth in the mouse model, *in vivo*. Intra-trachael infection of A/J mice with WT *L*. *pneumophila*, the *lamB* mutant, or the complemented mutant (*lamB/C*) was performed, as we described previously^43^. Within 10 days, 50% of the mice infected with WT or the complemented mutant had died. However, 100% of mice infected with the *lamB* mutant survived for the 10 days of the study (Fig. 6a). Analysis of bacterial burden in the lungs of surviving mice, at 24, 48, and 72 hrs showed that the *lamB* mutant had significantly decreased numbers of bacteria within the lungs, compared to the WT strain (Student *t-*test, *p* < 0.05) (Fig. 6b). The defective phenotype was completely recovered by complementation, indicating minimal loss of plasmid *in vivo* compared to *ex vivo* infection (Figs 4, 5). Histopathology on pulmonary biopsies taken at 12 and 24 hrs post-infection, with wild type *L*. *pneumophila* showed severe inflammatory infiltrates of mononuclear cells (Fig. 6c,d). In contrast, following challenge with the *lamB* mutant, minimal inflammatory infiltration into the alveolar, bronchial, or peribronical spaces was observed (Fig. 6c,d).

**Figure 7:**
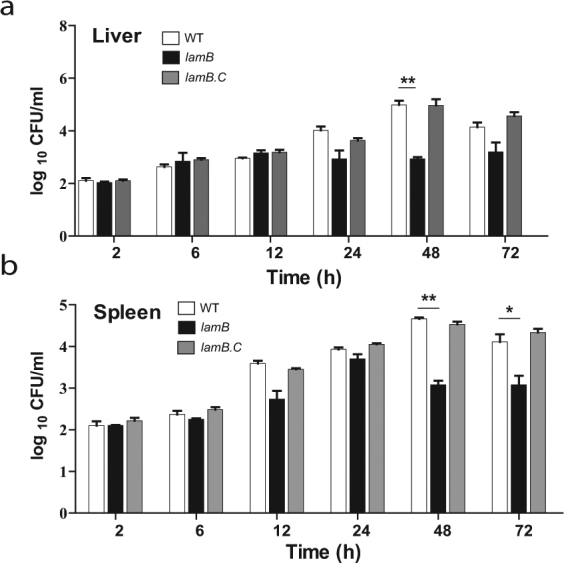
The role of LamB in dissemination of *L*. *pneumophila* in A/J mice. Mice were infected intra-trachaelly with 10^6^ CFUs of WT (white bars), the *lamB* mutant (black bars), or the completed, *lamB/*C (grey bars). Dissemination of the bacteria to the (**a**) liver and (**b**) spleen, as measured by CFU organ burden was assessed at the various time points.

The *lamB* mutant was less efficient in disseminating to the liver and spleen compared to the WT strain (Fig. 7a,b). At 48 hrs post-infection, there was a significant decrease in the amount of bacteria in the liver of mice infected with the *lamB* mutant compared to the WT strain (Student *t-*test, *p* < 0.01). Compared to the WT strain, fewer *lamB* mutants disseminated into the spleen at 48 hrs (Student *t-*test, *p* < 0.05) and 72 hrs (Student *t-*test, *p* < 0.01) post-infection compared to the WT strain. The reduced dissemination of the *lamB* mutant was completely restored upon complementation by *lamB*.

## Discussion

A “bipartite” metabolism has been used to describe the nutritional needs and metabolic regulation of *L*. *pneumophila*^4,47,50,79^. During early intracellular replication within human macrophages or amoebae, *L*. *pneumophila* relies on amino acids to generate carbon and energy from the TCA cycle^37,80^. Once amino acid levels become low, the bacteria undergo growth phase transition, switching from the replicative phase to the transmissive phase^81–84^. At this point, *L*. *pneumophila* increases uptake and utilization of glucose and converts it into the storage compound PHB^46,47^. Experiments with ^13^C-glucose demonstrated that glucose is used for *de novo* synthesis of amino acids and PHB during late stages of infection^39^. Additionally, labeling of glucose demonstrated a carbon flux from glucose to pyruvate via the Enter Doudoroff (ED) pathway but not the Pentose Phosphate Pathway (PPP)^39^. However, addition of excess exogenous glucose does not result in increased growth of the organism during any stage^46^. Therefore, generation of excess glucose in the host, as a source of carbon and energy, through degradation of polysaccharides by LamB is unlikely to support growth. Thus, it is surprising to identify a major role for LamB in intracellular growth, since *L*. *pneumophila* mainly utilizes amino acids for growth^85^. We speculate LamB is involved in processes other than nutritional virulence^85,86^.

Uptake of glucose is increased by *L*. *pneumophila* during post-exponential growth, most notably for the generation of the storage molecule, PHB^39,46,49^, and nutrient importers are important for intracellular growth of *L*. *pneumophila*^6,87^. Having large stores of PHB allows the organism to persist outside of the host for extended periods of time^51^.Transcriptomic studies in the human macrophage cell line THP-1 have demonstrated that expression of *lamB* is highest early in infection (8 hrs) rather than later (14 hrs), opposite of when the organism starts increasing consumption of glucose^46,88^. Faucher *et al*. also classified this gene as “highly induced in cells”^88^. LamB may be involved in increasing availability of glucose in the host ahead of when the organism prepares to utilize it during the late stages of infection. However, our data excludes that possibility. Increasing the availability of glucose with an amylase could allow *L*. *pneumophila* to generate more PHB, promoting long-term survival. It is possible that failure to store sufficient amounts of PHB by the *lamB* mutant results in an early defect in intracellular growth, due to the lack of a rapid influx of acetyl-CoA from reduced levels of PHB. Alternatively, since amylases are known to act on the glycosylation of proteins, LamB may be acting on the post-translational modification of host proteins to control processes important for replication, independent of nutrition or PHB storage^89^. This could allow the bacterium to evade some aspect of the innate host immune response necessary for successful intracellular replication. Future studies are aimed at identification of target(s) of LamB and how they contribute to infection. Considering LamB is unique to *L*. *pneumophila* and its loss causes complete defect in intracellular growth in macrophages and amoebae, and attenuation *in vivo*, it may contribute to the enhanced virulence of *L*. *pneumophila* and its prevalence as a disease-causing species compared to other *Legionella* species.

Bioinformatical analysis indicates that LamB does not contain a T2SS secretion signal, but it does however contain putative T4SS translocation signals^57,58,70^. However, through the CyaA reporter assay our data show that LamB is not translocated. Previous reports have shown effectors that not translocated as CyaA reporter assay, were actually translocated T4SS effectors^25,58^. This reporter could interfere with the translocation of LamB, like seen with other effectors^25^. Predicted strength of the translocation signal is not a definitive answer to whether a protein is translocated, low-scoring predicted effectors have been shown to be translocated by the Dot/Icm System and high-scoring proteins have been shown to not be translocated^58^. Lifshitz *et al*. identified LamB to be a high-scoring putative effector and in the same study tested 10 new high-scoring putative effectors, of which three were confirmed to not be translocated by the Dot/Icm system using the CyaA reporter assay, but LamB was not tested^58^. Despite being a high-scoring putative effector, LamB may not be translocated, as observed in our CyaA reporter assay. Loss of *lamB* does not affect *L*. *pneumophila*’s ability to grow *in vitro*, supporting the idea that LamB is likely secreted into the host cytosol or into the lumen of the LCV, but the mechanism remains to be determined.

In summary, we report an amylase essential for intracellular proliferation of *L*. *pneumophila* within the two evolutionarily distant hosts, human macrophages and amoebae. Given its uniqueness to *L*. *pneumophila*, LamB serves as an interesting enzyme that may contribute to the prevalence and virulence of *L*. *pneumophila* compared to other *Legionella* species.

## Materials and Methods

### Strains and cell lines

*L*. *pneumophila* strain AA100/130b (ATCC BAA-74) and the *dotA* T4SS-deficient mutant, were grown on Buffered Charcoal Yeast Extract (BCYE) agar, as we previously described^78^. To generate the isogenic mutant *lamB* (*lpg2528*), 2 kb flanking DNA on either side of *lamB*, was amplified using PCR with primers listed in Table S1. The resulting amplicon was cloned into the shuttle vector, pBCSk+, to generate pBCSK + *lamB*KO. To delete the entire gene of *lamB*, inverse PCR was employed using the primers listed in Table S1, resulting in pBCSK + *lamB*KO2. The kanamycin resistance cassette from the Ez-Tn5 transposon was amplified using primers listed in Table S1. The resulting PCR product was subcloned in pBSCK + *lamB*KO2 between the *lamB* flanking regions using standard molecular procedures, resulting in pBCSK + *lamB*KO3. This plasmid was introduced into *L*. *pneumophila* AA100/130b via natural transformation, as we previously described^90^. Natural transformants were recovered by plating on BCYE agar supplemented with 50 μg/ml kanamycin. To complement the *lamB* mutant, PCR was used to amplify the *lamB* gene and its upstream promotor region, using primers listed in Table S1, and subcloned into pBCSK + , generating pBCSK + *lamB*/C. Complement mutants of *lamB* with mutations in the catalytic pocket were made by substituting the amino acid for alanine, to generate pBCSK + lamBD193A, pBCSK + lamBD296A, and pBCSK + lamBE227A, using primers listed in Table S1. These plasmid was introduced into the *lamA* mutant, via electroporation, as previously described^91^. Complemented *lamB* mutants were selected on BCYE plates supplemented with 5 μg/ml chloramphenicol, resulting in the complemented strains, *lamB*/C, *lamB*/D193A, *lamB*/D296A, and *lamB*/E227A.

### Intracellular replication

For infection of cell monolayers, *L*. *pneumophila* strains were grown in BYE broth with appropriate antibiotic selection, at 37 °C with shaking, to post-exponential phase (OD_550nm_ 2.1–2.2). *A*. *polyphaga* was cultured in PYG media at 22 °C, experiments were performed in PY media at 35 °C, as previously described^78^. Glucose supplementation experiments were done in presence of 100 mM glucose in the media. Human monocyte-derived macrophages (hMDMs) were isolated from healthy donors and cultured in RPMI 1640, supplemented with 10% fetal bovine serum, as previously described^78,92^. All methods were approved and carried out in accordance to the University of Louisville Institutional Review Board guidelines and blood donors gave informed consent as required by the University of Louisville Institutional Review Board (IRB # 04.0358).

The wild type strain; the isogenic mutants, *dotA* and *lamB*; and complements *lamB*/C, *lamB*/D193A, *lamB*/E227A, and *lamB*/D296A were grown to post-exponential phase in BYE broth at 37 °C with shaking, prior to infection and used to infect hMDMs and *A*. *polyphaga*, as previously described^78,92^. A total of 1 × 10^5^ host cells were plated in 96 well plates and infected with *L*. *pneumophila* at an MOI of 10. Plates were centrifuged at 200 × g (5 mins), to synchronize infection. After 1 h, cells were treated for 1 h with gentamicin to kill extracellular bacteria, as previously described^78,92^. Over a 24 h time course, host cell were lysed with sterile water (hMDMs) or 0.02% v/v Triton X-100 (*A*. *polyphaga*). *L*. *pneumophila* CFUs were determined by plating serial dilutions onto BCYE agar.

### Bioinformatics analysis of LamB

Protein domain analysis was performed using NCBI’s Search for Conserved Domains (https://www.ncbi.nlm.nih.gov/Structure/cdd/wrpsb.cgi). Phylogenetic analysis was determined using amino acid sequences of LamB, with the Phyologeny.fr platform. Branch length was determined by aLRT^93,94^. Predicted structures were generated via I-TASSER (https://zhanglab.ccmb.med.umich.edu/I-TASSER/)^60^. Structures generated from I-TASSER were aligned using TM-align to generate a TM-score of structural similarity (https://zhanglab.ccmb.med.umich.edu/TM-align/).

### Translocation Assay

To assess translocation of LamB by *L*. *pneumophila* T4SS, during infection of host cells, an adenylate cyclase fusion^74^ was generated using standard biology techniques with primers listed in Table S1. A total of 1 × 10^6^ hMDMs were infected with wild type or *dotA* mutant *L*. *pneumophila* harboring plasmids expressing various adenylate cyclase fusions at an MOI of 20 for 1 h, as previously described^74,92^. Following infection, the cell monolayers were lysed and processed to assess cAMP concentration by ELISA using the Direct cAMP ELISA kit (Enzo) according to the manufacturer’s protocol and measure with a Synergy H1 microplate reader (BioTek).

### Amylase activity

To determine if LamB is a functional amylase, the *lamB* gene was cloned into the IPTG-inducible GST-fusion expression vector pGEX-6p-1 (Amersham) and expressed in *E*. *coli* BL21 using primers listed in Table S1. Additionally, residues within the predicted catalytic pocket were substituted to alanine using inverse PCR using primers listed in Table S1. *E*. *coli* cultures (5 ml) harboring either the empty vector, *lamB*, or the various catalytic inactive mutants were grown in LB broth at 37 °C with shaking until the OD_600nm_ reached 0.8. The cultures were spilt and one half was induced with 0.1 mM IPTG for 2.5 h at room temperature. One ml of each culture was pelleted by centrifugation and subjected to lysis with 0.5 ml buffer (0.1% v/v Triton X-100, 150 nM NaCl, 10 mM Tris pH7.5), containing protease inhibitors. Insoluble material was pelleted by centrifugation (16000 × g, 10 min, 4 °C) and the resulting supernatant was retained. Expression of fusion proteins was similar in all cultures (see Supplementary Fig. S3). To measure amylase activity, 25 μl of supernatant was analysed using an Amylase Assay Kit (Sigma), following the manufacturer’s instructions. This kit utilizes an artificial substrate, ethylidene-pNP-G7, which when cleaved by an amylase generates a colorimetric product detectable at 405 nm.

### Mouse model

For testing the virulence of the *lamB* mutant, specific pathogen-free, 6–8 weeks old A/J mice were used, as previously described^43,92^. Groups of 3 A/J mice, for each time point, were infected intratracheally with 1 × 10^6^ CFUs. At 2, 12, 24, 48, and 72 h after infection mice were humanely sacrificed and lungs, liver, and spleen were harvested and homogenized in sterile saline (5 ml) followed by cell lysis in distilled water. To determine CFUs, serial 10-fold dilutions were plated on BCYE agar and incubated at 37 °C. For histopathology, lungs of infected mice were fixed in 10% neutral formalin and embedded in paraffin. Serial 5 μm sections were cut, stained with haematoxylin and eosin (H&E), for light microscopy analysis. Twenty random high-powered fields (HPFs) were assessed to grade inflammation severity including alveolar and bronchial damage, as well as percentage of parenchyma involved. The histology assessment included the number of the mononuclear cells and percent of parenchyma involved by using modification of double-blind scoring method at a magnification of 40x, as we described previously^95^. The inflammation process was graded normal (score of 0), when there were 0–19 monocular cells infiltrates per HPF with no alveolar and bronchial involvement; mild (score of 1), for 20 to 49 cells per HPF, including mild damage of alveolar and bronchial regions; moderate (score of 2), for 50 to 99 cells per HPF with moderate alveolar and bronchial inflammation; or severe (score of 3), for 100 to 200 mononuclear cells per HPF with severe effacement of alveolar and bronchial regions. The murine lung section was examined in sagittal direction and percent of parenchyma involved was scored as 0 when no area was compromised. The involvement of the parenchyma was scored as 1 when up to 25% of the total area was occupied by inflammatory exudate, or scored as 2 when 26 to 50% of parenchyma area was occupied with inflammatory cells, and 3 if comprised of more than 51% of the total area. The total histology score was calculated as an average of individual criteria scores. Uninfected tissue was used as a baseline score. All the experimental procedures were in accordance with National guidelines and were approved by the Institutional Animal Care and Use committee (IACUC) at Faculty of Medicine, University of Rijeka.

### Data availability

All data generated or analysed during this study are included in this published article (and its Supplementary Information files).

## Electronic supplementary material

Supplementary Figures and Tables
