# Supplementary material for: A Legionella pneumophila amylase is essential for intracellular replication in human macrophages and amoebae
Source: Sci Rep. 2018 Apr 20;8:6340. doi: 10.1038/s41598-018-24724-1 (PMC5910436; doi:10.1038/s41598-018-24724-1)
Supplement: Supplementary file 1 — Supplementary Figures and Tables [file 41598_2018_24724_MOESM1_ESM.docx]

**Supplementary Figures and Tables for:**

**A *Legionella pneumophila* amylase essential for intracellular replication in human macrophages and amoebae**

Ashley Best^†1^, Christopher Price^†1^, Mateja Ozanic^2^, Marina Santic^2^, Snake Jones^1^, and Yousef Abu Kwaik*^1,3^

^1^Department of Microbiology and Immunology, College of Medicine, University of Louisville, Louisville, KY, ^2^Department of Microbiology and Parasitology, Faculty of Medicine, University of Rijeka, Rijeka, Croatia, ^3^Center for Predictive Medicine, University of Louisville, Louisville, KY

*To whom correspondence should be addressed: [abukwaik@louisville.edu](mailto:abukwaik@louisville.edu)


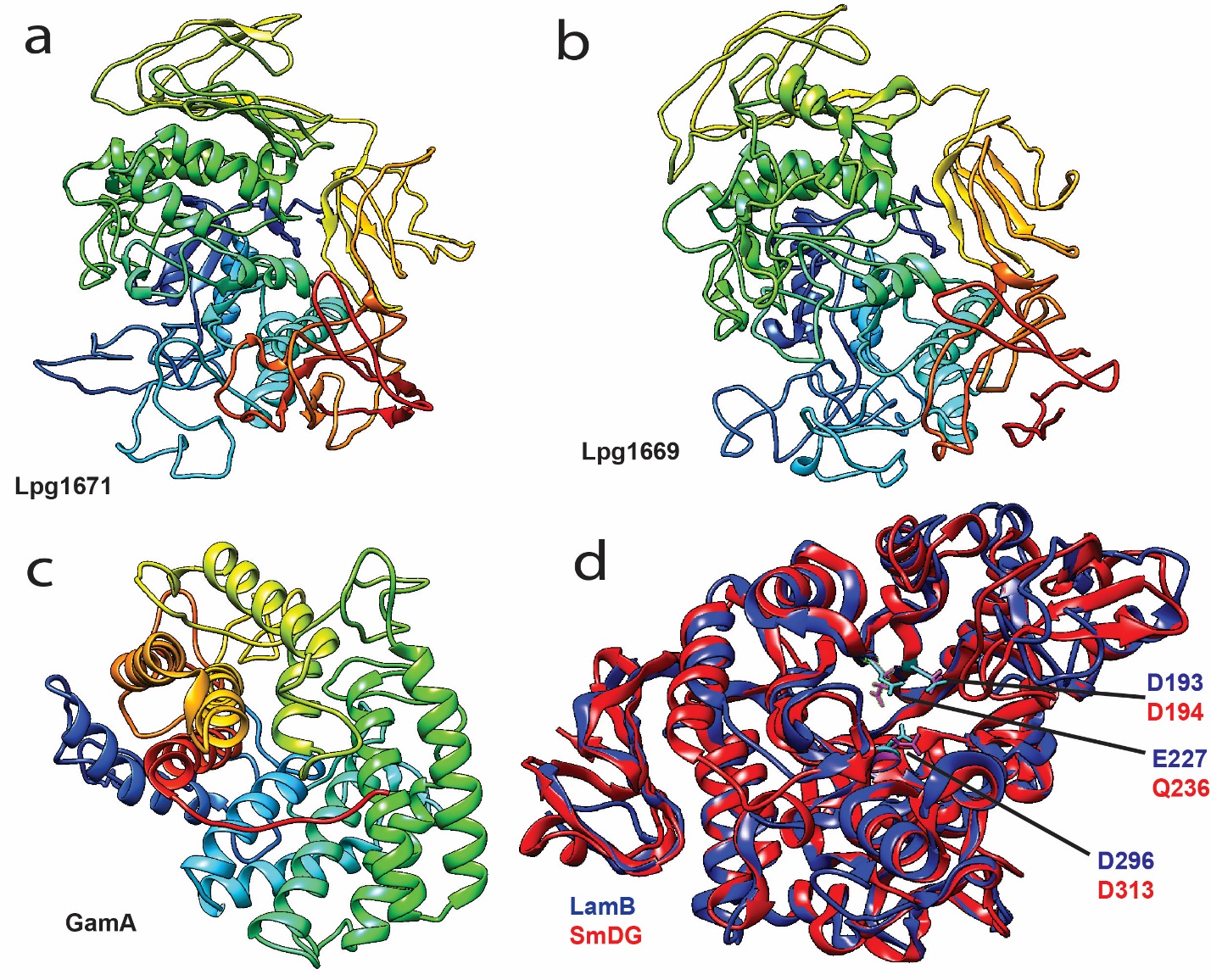


**Figure S1. Putative structure of amylases in *L. pneumophila*.** Two additional putative amylases, A) Lpg1672 and B) Lpg1669, were identified in *L. pneumophila* by domain sequence homology. C) The *L. pneumophila* GamA has been previously described as an amylase and substrate of type-II secretion. D) The predicted structure of LamB is similar to the crystalized structure of *Streptococcus mutans* dextran glucosidase, which share a conserved alpha amylase catalytic domain (NCBI domain: cl07893) and catalytic residues that are highlighted.


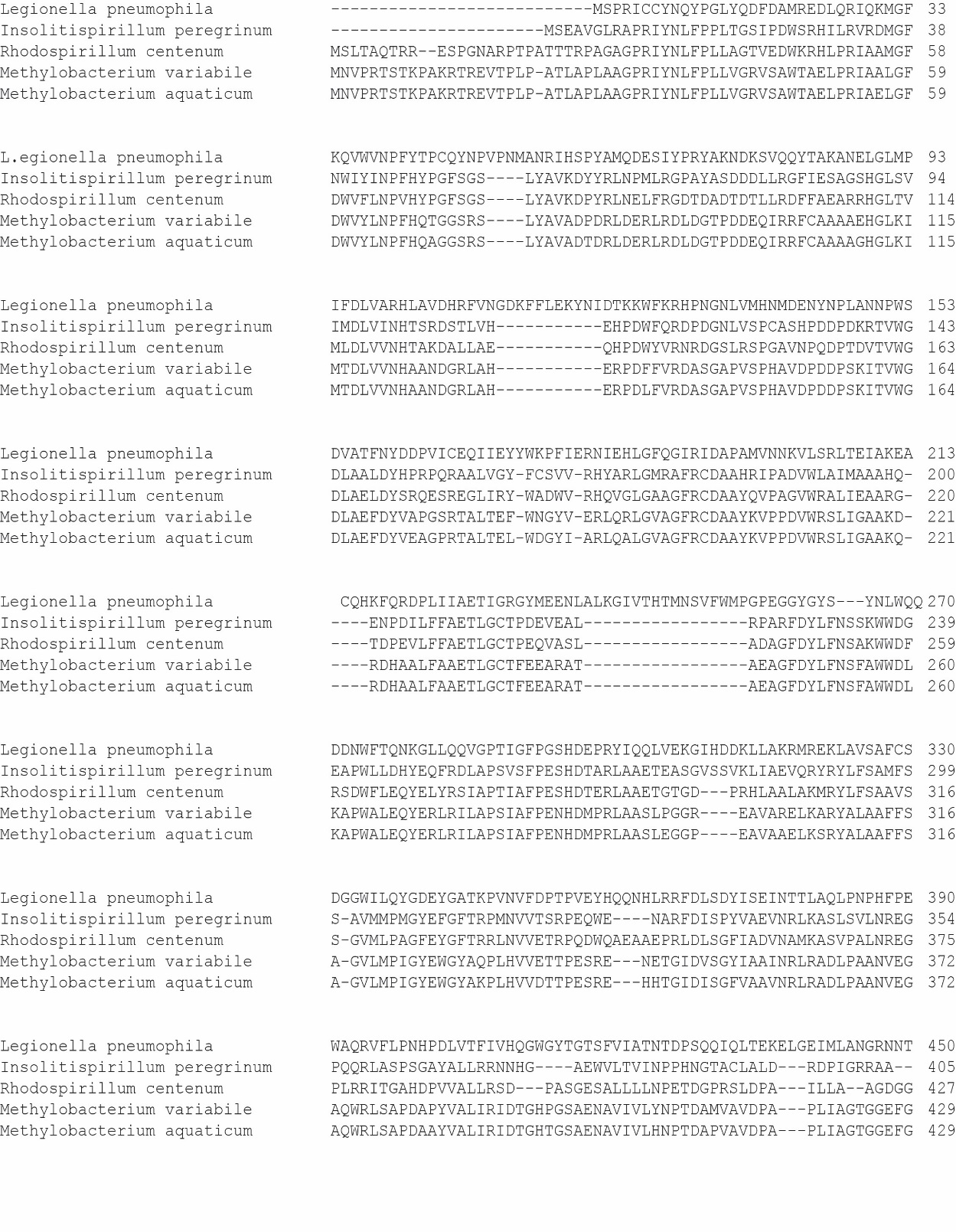


**Figure S1. LamB-like proteins in intra-amoebal and environmental pathogens.** Amino acid sequence homology of LamB shows alignment to other intra-amoebal and aquatic organisms like; *Insolitispirillum peregrinum*, isolated from a pond [^79^](#_ENREF_79)^,^[^80^](#_ENREF_80); *Rhodospirillum centenum*, isolated from the edge of a thermal spring [^81^](#_ENREF_81); *Methylobacterium variabile,* isolated from a drinking water [^82^](#_ENREF_82); and *Methylobacterium aquaticum*, also isolated from drinking water and capable of surviving and lysing amoebae [^83^](#_ENREF_83)^,^[^84^](#_ENREF_84), but not other known pathogens.

**
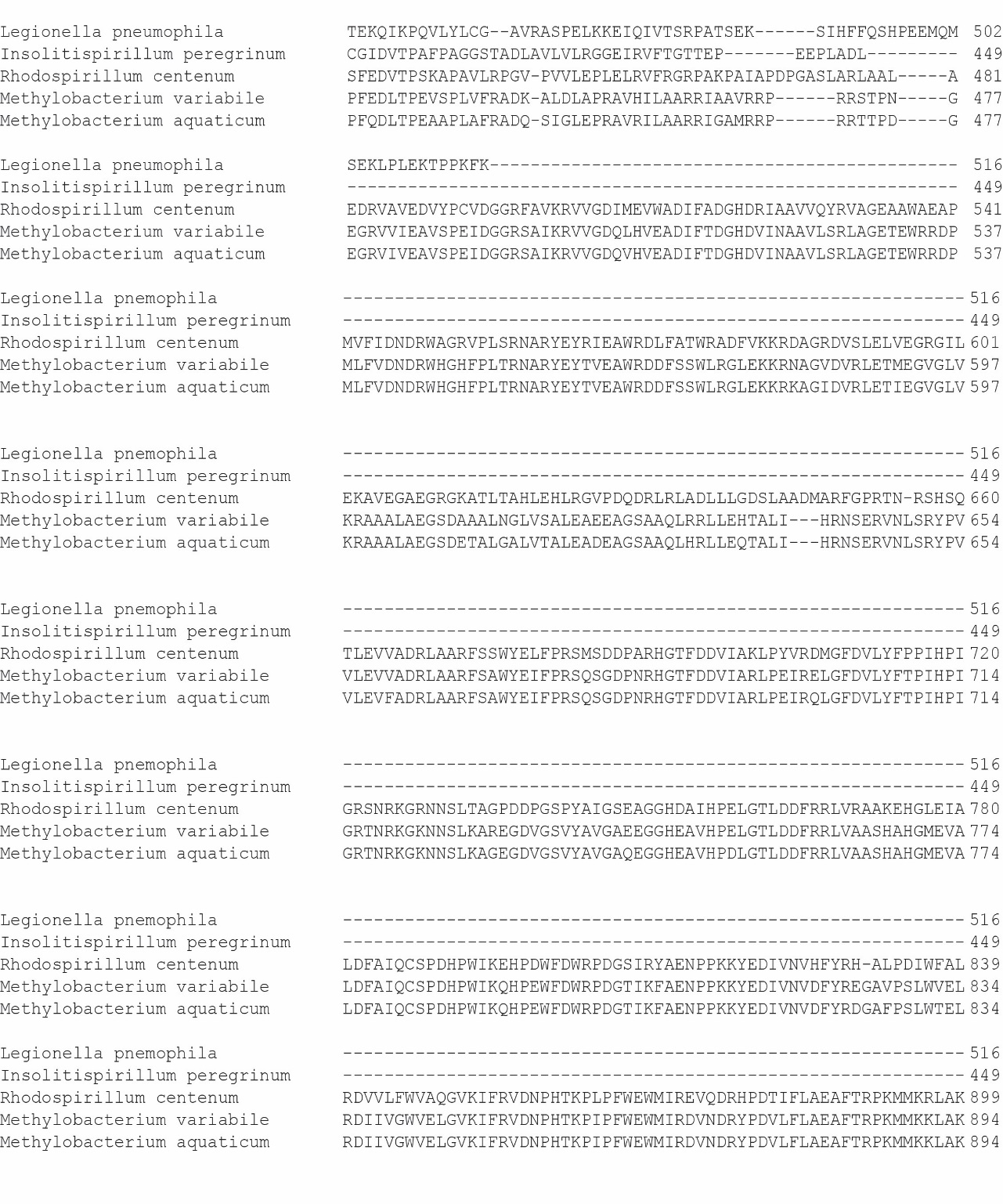
**

**Figure S1. LamB-like proteins in intra-amoebal and environmental pathogens.** Amino acid sequence homology of LamB shows alignment to other intra-amoebal and aquatic organisms like; *Insolitispirillum peregrinum*, isolated from a pond [^79^](#_ENREF_79)^,^[^80^](#_ENREF_80); *Rhodospirillum centenum*, isolated from the edge of a thermal spring [^81^](#_ENREF_81); *Methylobacterium variabile,* isolated from a drinking water [^82^](#_ENREF_82); and *Methylobacterium aquaticum*, also isolated from drinking water and capable of surviving and lysing amoebae [^83^](#_ENREF_83)^,^[^84^](#_ENREF_84), but not other known pathogens.

**
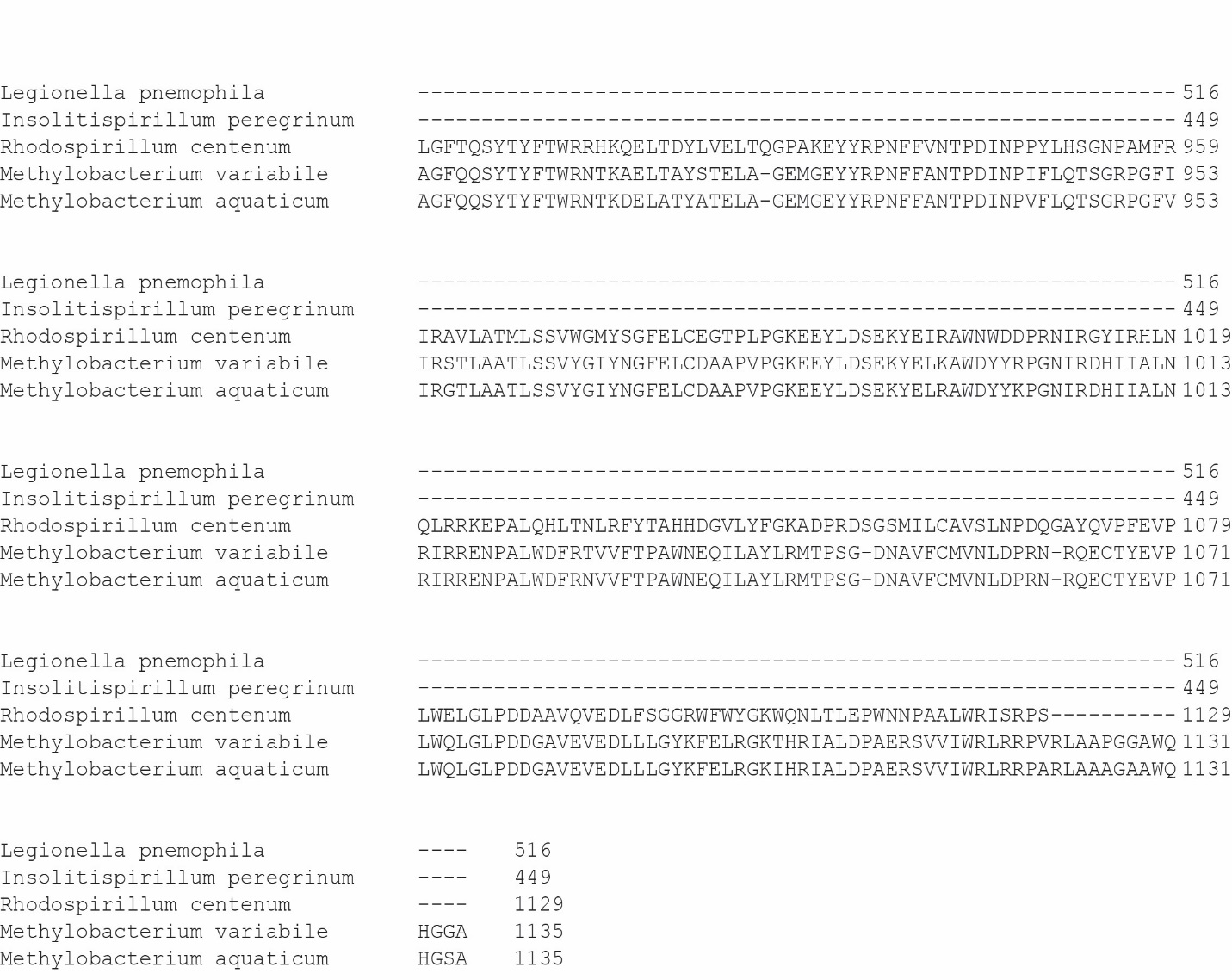
**

**Figure S1. LamB-like proteins in intra-amoebal and environmental pathogens.** Amino acid sequence homology of LamB shows alignment to other intra-amoebal and aquatic organisms like; *Insolitispirillum peregrinum*, isolated from a pond [^79^](#_ENREF_79)^,^[^80^](#_ENREF_80); *Rhodospirillum centenum*, isolated from the edge of a thermal spring [^81^](#_ENREF_81); *Methylobacterium variabile,* isolated from a drinking water [^82^](#_ENREF_82); and *Methylobacterium aquaticum*, also isolated from drinking water and capable of surviving and lysing amoebae [^83^](#_ENREF_83)^,^[^84^](#_ENREF_84), but not other known pathogens.

**
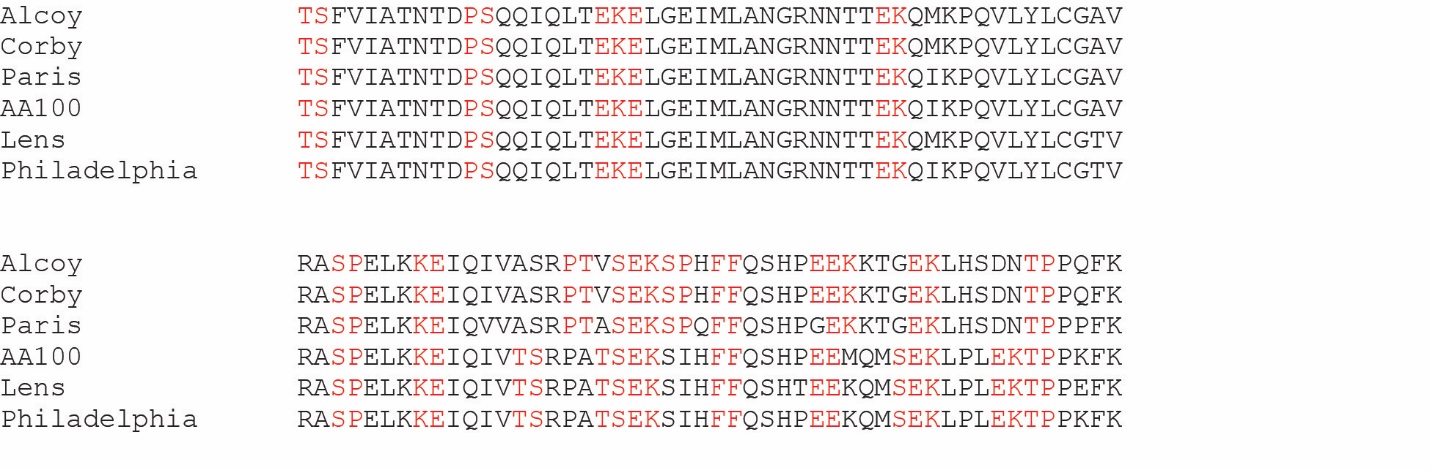
**

**Figure S2. The C terminal 100 amino acids of LamB.** Bi-residues identified to be heavily enriched in the last 100 amino acids of T4SS effectors are highlighted in red for multiple strains of *L. pneumophila* LamB and *L. steigerwaltii* LamB. Seventeen bi-residues are found in LamB of the AA100 strain.

**
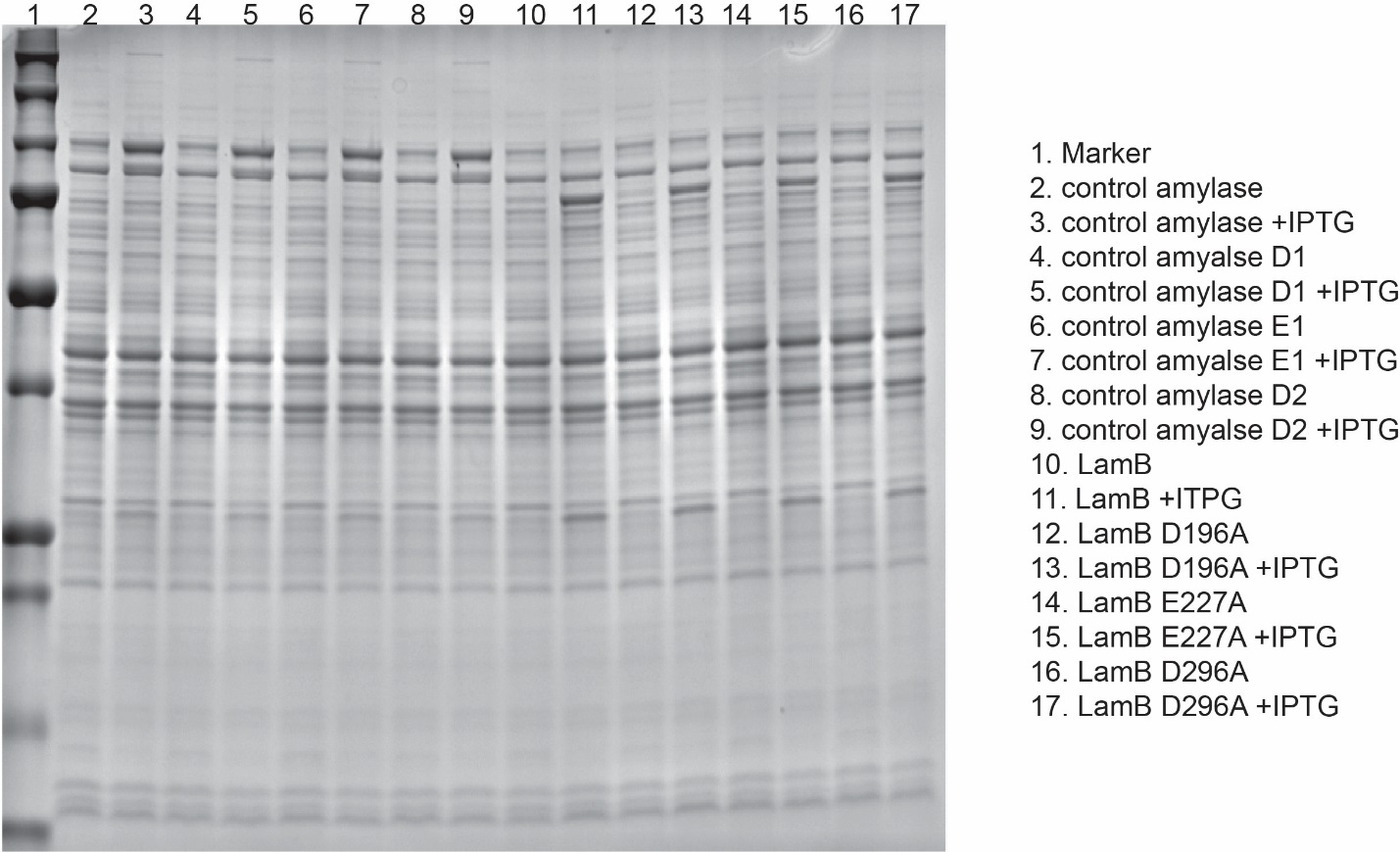
**

**Figure S3. Induction of GST-LamB fusions in *E. coli*.** *E. coli* BL12 harboring GST-LamB, GST-LamB D193A, GST-LamB E227E, or GST-LamB D296 constructs were grown to an OD_600_ 0.8 in LB broth before induction with 0.1mM IPTG at 37°C for 2.5h. Coomassie stain of uninduced and induced cultures shown.


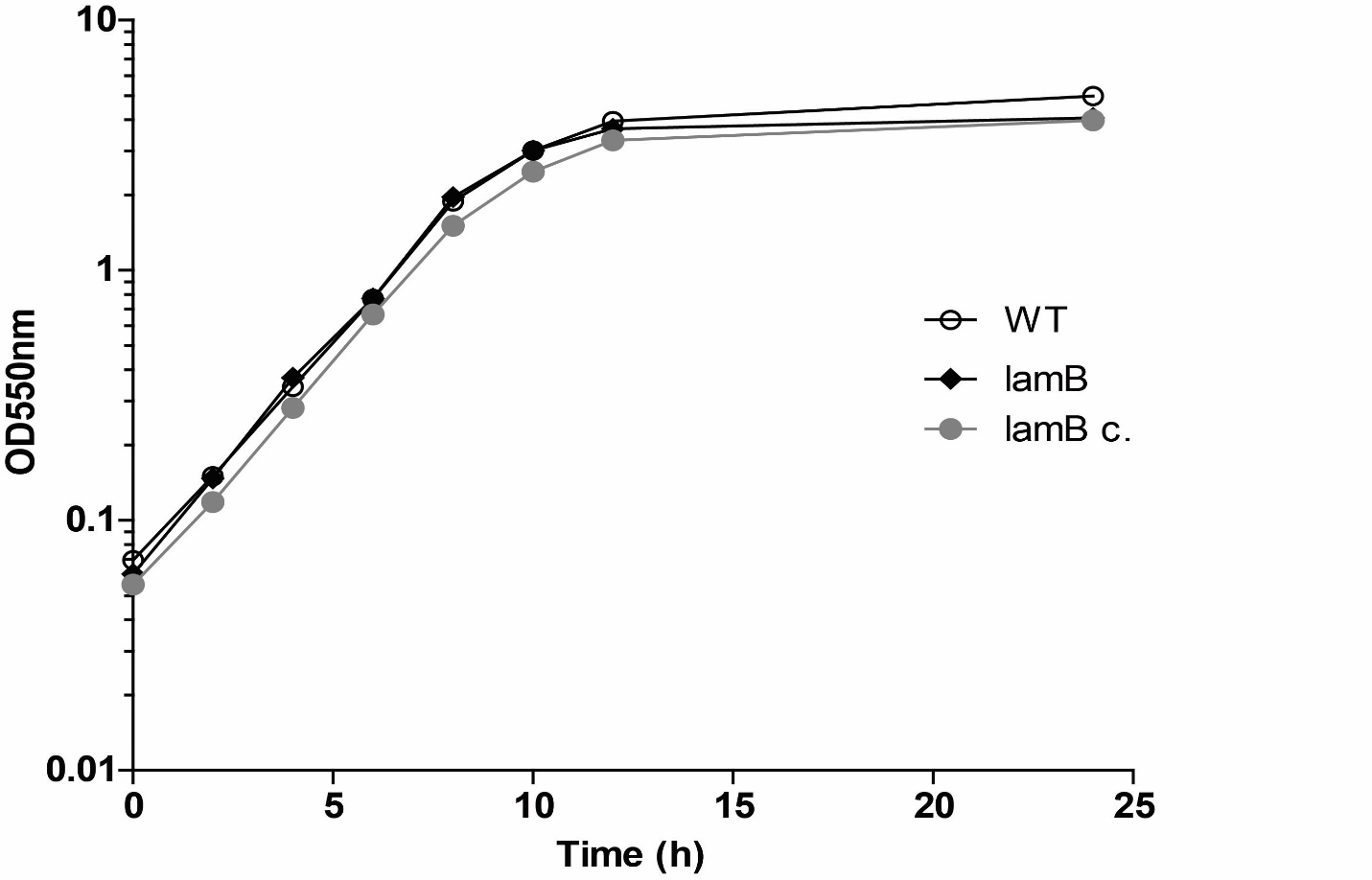


**Figure S4. Growth of mutants *in vitro.*** Overnight cultures of WT, Δ*lamB*, or catalytic mutants in BYE broth were grown overnight at 37°C then diluted to OD_550_ 0.05 and grown at 37°C for 24h. Growth rates were determined by measuring optical density at 550nm every two hours, for 12, then again at 24h post-inoculation. Data representative of three independent experiments.

**
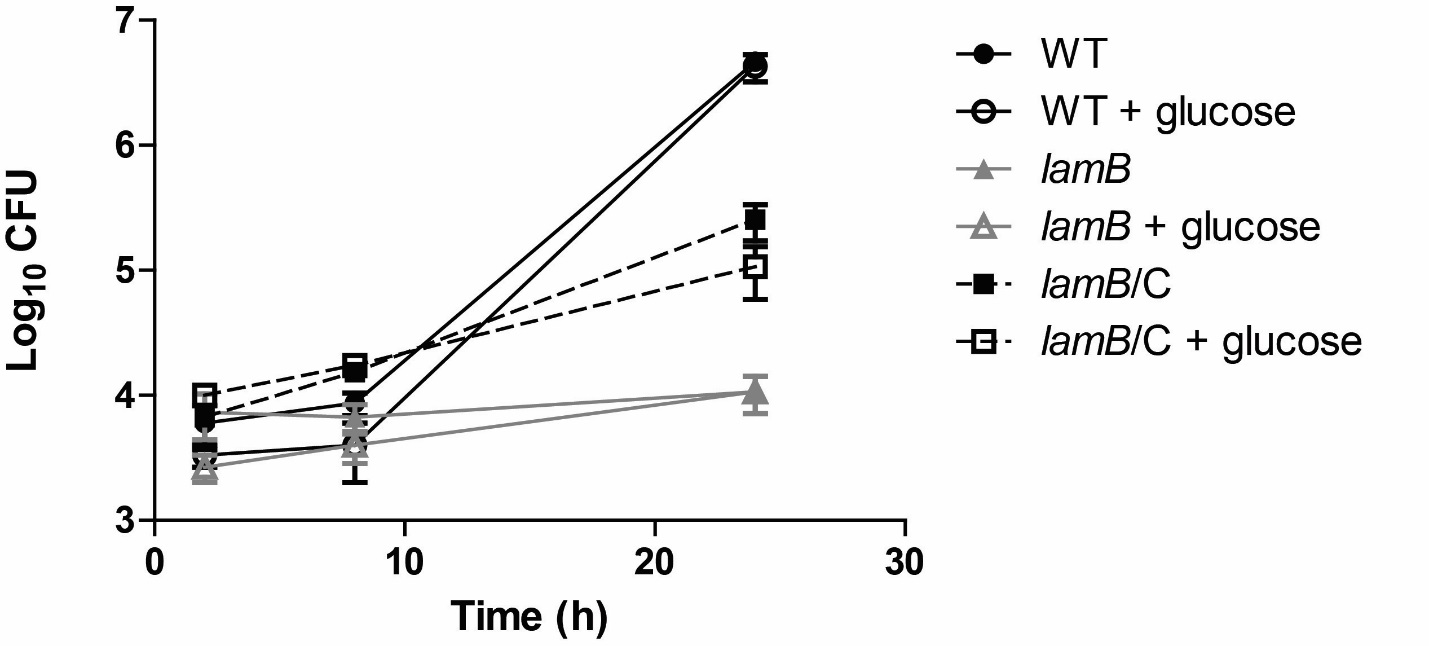
**

**Figure S5. Supplementation of *A. polyphaga* with glucose does not compensate for the loss of *lamB*.** Intra-vacuolar replication of the WT strain, the *lamB* mutant*,* and complemented *lamB* mutant (*lamB/*C) in *A. polyphaga* was determined upon glucose supplementation. The number of CFUs was determined at 2, 8, and 24h post-infection. Data points represent (mean CFUs ± SD, n=3) and are representative of two independent experiments.

**Table S1. Primers used in this study**

| Primers | Sequence |
| --- | --- |
| lamB-KO F | GGATCCTAATGTCTTTATTACTTCAC |
| lamB-KO R | GTCGACTTTTCGTATCAAATAAACTA |
| lamB inverse F | TTTACCTGCAGGATAATAATATTTACCGGCT |
| lamB inverse R | TTTATTGATGCAGATCGCTGACTTCTCAATTTC |
| Pcr confirm KO F | GAACAGAAATTGAGAAGTCAGC |
| PCR confirm KO R | TATAAATGCAATATAGCCGGTAAATATTA |
| ­lamB COMP F | CTCGAGAGTAAATATGATGCCCATAA |
| lamB COMP R | GGATCCTTATTTAAATTTAGGTGGTGTT |
| Kan F, R | /5Phos/CTGTCTCTTATACACATCTCAA |
| lamB CYA F | GGATCCTTATGTCTCCACGGATTTGTTGC |
| lamB CYA R | AAGCTTTTATTTAAATTTAGGTGGTG |
| lamB-GST fusion (pGEX) F | GGATCCATGTCTCCACGGATTTGTTG |
| lamB-GST fusion (pGEX) R | GTCGACTTATTTAAATTTAGGTGGTG |
| lamB D193A F | /5Phos/AGCAATACGTATTCCCTG |
| lamB D193A R | /5Phos/GCCCCAGCCATGGTAAAT |
| lamB E227A F | /5Phos/TGCTGCAATAATAAGTGG |
| lamB E227A R | /5Phos/ACCATAGGCAGAGGTTAT |
| lamB D296A F | /5Phos/AGCATGAGATCCAGGAAA |
| lamB D296A R | /5Phos/GAGCCACGCTATATTCAG |
